# Ligand-induced dynamics of heterotrimeric G protein-coupled receptor-like kinase complexes

**DOI:** 10.1371/journal.pone.0171854

**Published:** 2017-02-10

**Authors:** Meral Tunc-Ozdemir, Alan M. Jones

**Affiliations:** 1 Department of Biology University of North Carolina at Chapel Hill, Chapel Hill, North Carolina, United States of America; 2 Department of Pharmacology, University of North Carolina at Chapel Hill, Chapel Hill, North Carolina, United States of America; National Taiwan University, TAIWAN

## Abstract

**Background:**

Arabidopsis, 7-transmembrane Regulator of G signaling protein 1 (AtRGS1) modulates canonical G protein signaling by promoting the inactive state of heterotrimeric G protein complex on the plasma membrane. It is known that plant leucine-rich repeat receptor–like kinases (LRR RLKs) phosphorylate AtRGS1 *in vitro* but little is known about the *in vivo* interaction, molecular dynamics, or the cellular consequences of this interaction.

**Methods:**

Therefore, a subset of the known RLKs that phosphorylate AtRGS1 were selected for elucidation, namely, BAK1, BIR1, FLS2. Several microscopies for both static and dynamic protein-protein interactions were used to follow *in vivo* interactions between the RLKs and AtRGS1 after the presentation of the Pathogen-associated Molecular Pattern, Flagellin 22 (Flg22). These microscopies included Förster Resonance Energy Transfer, Bimolecular Fluoresence Complementation, and Cross Number and Brightness Fluorescence Correlation Spectroscopy. In addition, reactive oxygen species and calcium changes in living cells were quantitated using luminometry and R-GECO1 microscopy.

**Results:**

The LRR RLKs BAK1 and BIR1, interact with AtRGS1 at the plasma membrane. The RLK ligand flg22 sets BAK1 in motion toward AtRGS1 and BIR1 away, both returning to the baseline orientations by 10 minutes. The C-terminal tail of AtRGS1 is important for the interaction with BAK1 and for the tempo of the AtRGS1/BIR1 dynamics. This window of time corresponds to the flg22-induced transient production of reactive oxygen species and calcium release which are both attenuated in the *rgs1* and the *bak1* null mutants.

**Conclusions:**

A temporal model of these interactions is proposed. flg22 binding induces nearly instantaneous dimerization between FLS2 and BAK1. Phosphorylated BAK1 interacts with and enables AtRGS1 to move away from BIR1 and AtRGS1 becomes phosphorylated leading to its endocytosis thus leading to de-repression by permitting AtGPA1 to exchange GDP for GTP. Finally, the G protein complex becomes dissociated thus AGB1 interacts with its effector proteins leading to changes in reactive oxygen species and calcium.

## Introduction

Heterotrimeric G proteins couple extracellular signals to cytoplasmic changes in multiple abiotic [1–4] and biotic stress responses [5–8], and developmental cues. A growing body of evidence indicates that signal specificity is achieved by Leucine-Rich Repeat Receptor-Like Kinases (LRR-RLK) and G protein complexes in related pathways [9–16]. In Arabidopsis, the heterotrimeric G protein complex is composed of one canonical Gα subunit (AtGPA1), one Gβ subunit (AGB1) and one of three Gγ (AGG1, AGG2 and AGG3) subunits [17]. The canonical Gα subunit AtGPA1 self-activates through spontaneous GDP/GTP exchange without G-protein coupled receptors (GPCR) unlike its counterparts in animals [18]. Once Gα is activated, it interacts with downstream target proteins [19]. AtRGS1, an N-terminal seven-transmembrane (7TM) domain fused to a Regulator of G-protein Signaling (RGS) protein, fosters the G protein complex into the inactive state by accelerating intrinsic GTP hydrolysis activity of Gα [20]. Phosphorylation of AtRGS1 is required for its endocytosis similar to GPCRs [21,22]. Endocytosis of AtRGS1 leads to activation of the Gα protein allowing spontaneous nucleotide exchange and sustained activation [20]. In Arabidopsis, glucose-mediated phosphorylation occurs by three WITH NO LYSINE (WNK) kinases [20,23] and by several LRR-RLK at the same critical serine residue [16]. BAK1 phosphorylates AtRGS1 in response to Pathogen-Associated Molecular Pattern (PAMP) flg22 and induces AtRGS1 endocytosis [16]. In *Glycine max*, a 7TM-RGS paralog, GmRGS1, is phosphorylated by a lysine-motif family of RLKs, designated Nod factor receptor 1 [9]. Hence, phosphorylation and endocytosis of AtRGS1 is the crux of ligand-dependent signal modulation of heterotrimeric G proteins. In this study, a set of AtRGS1 LRR-RLK partners and their dynamics in canonical G signal transduction is used to model ligand-induced dynamics of heterotrimeric G protein-coupled receptor-like kinase complexes. The RLK ligand flg22 was chosen for this model because G protein signaling is directly activated by flg22 through FLS2 with its co-receptor BAK1 [16]. ROS was chosen as the signaling output because G proteins are involved in ROS production triggered by flg22 [6,24]. In addition to the sole canonical Gα subunit, Arabidopsis has three atypical Gα subunits called extra-large GTP-binding proteins (XLG1, XLG2, and XLG3) that bind Gβγ dimers *in vitro*. XLGs consist of an N-terminal domain of unknown function and a C-terminal Gα- like domain [25,26]. Both structural and experimental evidence indicate that XLG proteins bind Gβγ dimers but have lost the guanine nucleotide dependency [27]. Moreover, XLGs do not bind to AtRGS1 [27], therefore the XLG role may be indirect such as sequestering AGB1. The non-canonical heterotrimeric G protein complex (XLG2/3, AGB1, and AGG1/2) interacts with the FLS2-BIK1-RbohD complex and flg22 leads to phosphorylation of the amino terminal domain of XLG2 by BIK1. The phosphorylated G protein dissociates from the receptor complex and regulates RbohD [13]. Therefore, the atypical G protein complex functions downstream of the RLK FLS2 to regulate the PAMP-triggered oxidative burst.

Here, the physical and co-functional relationship of a subfamily of the LRR RLKs with AtRGS1 is analyzed by following protein-protein interactions over time, subcellular space, and concentration of flg22. Finally, we integrate our observations with the flg22-induced changes evoked by the previously-described atypical G protein pathway [13,26].

## Materials and methods

### Plant materials and flg22-induced root growth inhibition assay

*Arabidopsis thaliana* (Arabidopsis) Col-0 and T-DNA insertion null mutants *rgs1*-2 (SALK_074376.55.00) [28], *bak1*-4 (SALK_116202) [29], *fls2* (SAIL_691_C4) [30] *rgs1*-2*/ agb1*-2, *rgs1*-2*/ gpa1*-4, *rgs1*-2*/ agb1*-2/ *gpa1*-4 plants were grown in soil under fluorescent lights [12 h light (60 μEinstein/m^2^/s) and 12 h dark] at 23°C for ROS assays. *Nicotiana benthamiana* plants were maintained in a plant growth room at 26°C with a 16 h light (120 μEinstein/m^2^/s) and 8 h dark photoperiod for BiFC and FRET experiments.

### Live cell imaging with R-GECO1

Leaves of seedlings expressing the R-GECO1 calcium reporter were grown on ¼ MS agar plates without sugar for two weeks (12 h light [120 μEinstein/m^2^/s] and 12 h dark photoperiod) and imaged using a Zeiss LSM710 confocal laser scanning microscope equipped with a Plan-NeoFluor 20×/0.5 objective and a C-Apochromat 40×/1.20 water immersion objective. R-GECO1 was excited using a 560-nm diode laser. Fluorescence emission was detected between 620 and 650 nm by a photomultiplier detector. The digital images were analyzed with ImageJ.

### ROS analyses

The flg22-induced ROS burst was measured according to Chung and coworkers [31]. Briefly, leaf discs from ~5 week-old plants were placed singly into a 96-well plate with 250 μl of water per well. After overnight incubation, the water was replaced with 100 μl of reaction mix (17 μg/ml of Luminol (Sigma), 10 μg/ml of Horseradish Peroxidase (HRP; Sigma), and 1 μM flg22). Luminescence was measured immediately with 1 s integration and 2 min interval over 40 to 50 min using a SpectraMax L (Molecular Device).

### Transient expression in *N*. *benthamiana* for Förster Resonance Energy Transfer (FRET) analyses and Bimolecular Fluorescence Complementation (BiFC)

Transient expression for BiFC was performed according to the protocol described by Tunc-Ozdemir and coworkers [32]. nYFP- and cYFP-tagged proteins as described previously by Urano and coworkers [20] were generated by subcloning the genes of interest into pENTR/D-TOPO then recombining into one or more of the BiFC vectors pBatTL–sYFP-N or pBatTL–sYFP-C (for C-terminal tagged nYFP and cYFP halves, respectively) and pCL112_JO or pCL113_JO (for N-terminal tagged nYFP and cYFP halves, respectively). The mitochondrial RFP marker; Mt-rk obtained from the ABRC (CD3-991)) was used as an internal positive transformation control. On the 4^th^ to 6^th^ day post-infiltration, confocal images of BiFC samples were acquired using a Zeiss LSM710 confocal laser scanning microscope equipped with a C-Apochromat 40X/1.20NA water immersion objective. A 489-nm diode laser was tuned to excite YFP and emission was detected at 526–563 nm by a photomultiplier tube detector. mt-Rk data were collected from 583–622 nm range after the sample was excited with a 561-nm diode laser. Transient expression in *N*. *benthamiana* for FRET was performed following precautions for FRET analysis described by Tunc-Ozdemir and co-workers [32]. Briefly, Agrobacterium carrying a binary plasmid encoding either AtRGS1-YFP, AtRGS1ΔCt-YFP, BAK1-CFP, BIR1-CFP, GPA1-CFP or P19, which is a viral RNA silencing suppressor [33] were grown in 3 ml LB medium with 50 μg ml^−1^ rifampicin, 50 μg ml^−1^ gentamycin, and 100 μg ml^−1^ kanamycin (or spectinomycin based on the plasmid selection marker) overnight in 28°C incubator at 220 rpm. Fifty ml fresh LB with 50 μg ml−1 rifampicin, 50 μg ml−1 gentamycin, 100 μg ml^−1^ kanamycin/spectinomycin, and 20 μM acetosyringone was inoculated with 200 μl of overnight culture. Bacteria cells were then harvested by centrifugation at 5,900 relative centrifugal force for 15 min 20°C–24°C after the culture was grown overnight at 28°C (220 rpm). Cells were resuspended and diluted in infiltration buffer (10 mM MgCl2, 10 mM 2-(N-morpholino) ethanesulfonic acid and 200 μM acetosyringone) buffer. Agrobacterium carrying plasmids of interest were cultured to an OD_600_ of 1.0 along Agrobacterium expressing the gene silencing suppressor protein P19, then mixed in a 3:1 ratio and infiltrated into the abaxial side of 4–5 week-old *N*. *benthamiana* leaves with a needleless syringe. For acceptor photobleaching, 514-nm and a 458-nm argon lasers were tuned to excite YFP (acceptor) and CFP (donor) respectively. Acceptor and donor channels’ emissions were detected within the range of 516–596, 460–517 nm respectively. Region of interests were scanned 5 times (each for 100 iterations) using a 514-nm argon laser line at 100% intensity with a pinhole diameter set to 1.00 airy units to selectively induce acceptor photobleaching until it reached ~20–30% of its initial value. FRET efficiency was then estimated via Zen Software (http://www.zeiss.com/microscopy/en_de/downloads/zen.html). Regions of interest that show either decreased donor fluorescence intensity or no change in acceptor fluorescence intensity after bleaching were excluded from the calculations.

### Mesophyll protoplast transformation

Protoplasts from Arabidopsis Col–0 plants were generated as described previously [34] and transformed with BIR1-cYFP and AtRGS1-nYFP or AtRGS1-cYFP and AtRGS1-nYFP pairs by the method described by Abel and Theologis [35]. Two days after transformation, YFP signal was detected using a confocal laser-scanning microscope (Zeiss LSM 710 Duo, http://www.zeiss.com/) with a 489-nm diode laser that was tuned to excite YFP and a photomultiplier tube detector detecting emission at 526–563 nm. Chloroplast autofluorescence was detected from 689 to 758 nm. All images were processed using Zen2009 confocal software (Carl Zeiss, http://www.zeiss.com/microscopy/en_us/downloads/zen.html).

### Cross molecular number and brightness (Cross N&B) analysis

The binding ratio of protein-protein interaction was determined with Cross N&B analysis, which is based on correlation of fluorescence intensity fluctuation measured from microscope images. Cross N&B analysis was performed as described by Clark et al., and others [36,37]. Images of leaf disks transiently expressing AtRGS1 YFP and BIR1 CFP or BAK1 CFP were obtained using a Zeiss LSM880 confocal microscope (http://www.zeiss.com/) equipped with a Multiline Argon laser (458-nm line selected for CFP excitation and 514-nm line selected for YFP excitation). Detection of CFP emission was configured at 463–510 nm using a highly sensitive and stable GaAsP detector and detection of YFP emission was configured at 517–550 nm using a PMT (photomultiplier tube) detector. Image data was obtained from a time series of 100 images (no pause between time points) collected using a 256x256 pixel frame with pixel size set to 0.1 μm and a pixel dwell time of 4.12 μsec. Since each pixel was collected at a different time it generated spatio-temporal information that was used to calculate the average brightness of the particle (the ratio of the variance to the average intensity at each pixel) and Cross N&B using SimFCS Software Analysis [38]. The brightness of each protein was compared to the cross-brightness at each pixel and a brightness stoichiometry histogram was generated. A large, positive cross-brightness indicates that the two proteins bind at that pixel in the image. Given that the sensitivity of the detectors used for YFP and CFP channels are different, the S-factor, an imaging parameter that shifts the brightness of the image, such that the immobile fraction has a brightness value of 1, for the calibration of the two channels were significantly different (S factor for YFP channel 1.3; whereas S factor for CFP is 4) resulting in larger uncertainty for the CFP channel.

### *in vitro* co-precipitation assay

The *in vitro* pull-down assays were performed as described by Lee and coworkers [39]. Briefly, 10 μg of purified BIR1 kinase domain (between 303–620 aa)-MBP proteins bound to amylose resin were incubated with 2 μg of either AtGPA1, Gβγ or AtRGS1 box in binding buffer (50 mM Tris-HCl, pH 7.5, 100 mM NaCl, and 0.1% Triton X-100) at 4°C for 4 hrs. Samples were washed three times with washing buffer (50 mM Tris-HCl, pH 7.5, 200 mM NaCl, and 0.1% Triton X-100) then eluted using 5× SDS sample buffer with boiling for 5 min and blotted. The blot was probed with either anti-AtGPA1 (directed against the C-terminal peptide of GPA1 encoded by a 0.9 kB fragment of GPA1 cDNA [40]), anti-AGB1 (directed against a peptide consisting of the 18 N-terminal residues (Thr-14 to Leu-31) of AGB1), or anti-AtRGS1 antibody (directed against AtRGS1 amino acids 284–459).

## Results

### AtRGS1 is required for full RLK-dependent ROS production, and calcium signaling

**Fig 1:**
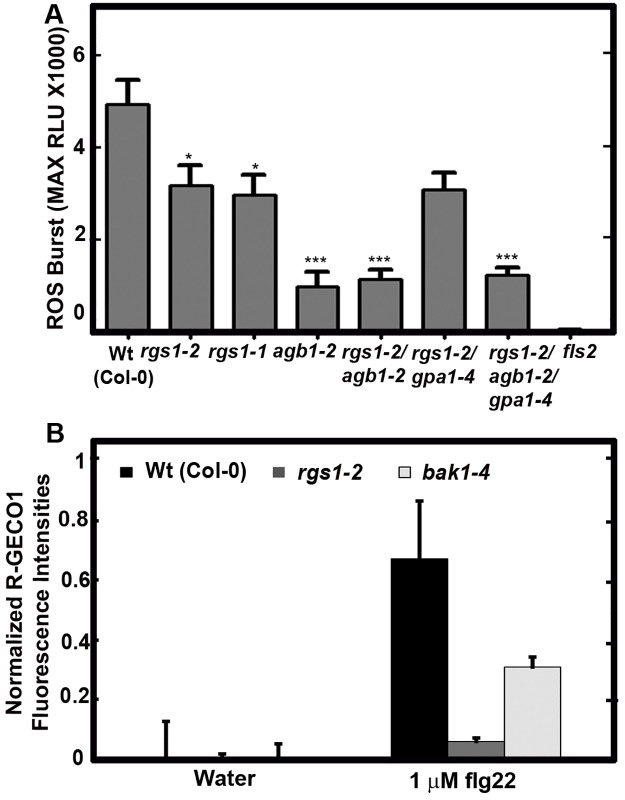
AtRGS1 functions in an early step in ROS production. (**A**) flg22-induced, FLS2-dependent ROS production is greatly attenuated in the *rgs1* null mutant. ROS, reported as Relative Luminescence Units (RLU) in leaf disks treated with flg22 (1 μM) from 5-week-old seedlings was measured over time (S1 Fig) as described in Materials and Methods. The graph shows the maximum relative light units (Max RLU) taken at the peak of ROS production and is plotted for all genotypes. Error bars are SEM and sample size *n* = 6–15. *, P<0.05, *** P<0.001 by Student's t-test compared to wild type (wt). (**B**) flg22-induced Ca^2+^ signal requires AtRGS1. Fluorescence intensity changes of R-GECO1 in ~50 regions of interests in wild type, *rgs1*-2 and *bak1*-4 plants. Fractional fluorescence changes (ΔF/F) for R-GECO1 were calculated from background corrected intensity values of R-GECO1 as (F − F0)/F0, where F0 represents the average fluorescence intensity of the baseline of a measurement of each genotype. Error bars are standard deviations.

One of the most rapid known events of the plant immune response is the ROS burst, beginning within tens of seconds of recognition of the PAMP, peaking around 10 minutes then returning to base line with a decay of ~3% maximum response per minute in Arabidopsis (e.g. S1 Fig but also demonstrated by many others). Because of the involvement of G protein complexes in PAMP-induced ROS production [6,24] and because AtRGS1 modulates the activation state of the canonical Gα subunit, we quantitated flg22-induced ROS production in the *rgs1* null mutants. Early ROS production in Arabidopsis leaves in response to flg22 was quantitatively measured via a luminol-based assay. The peak of ROS production was attenuated ~36 to 40% (*P* value < 0.001; Student's t-test is used to compare to wild type) at the maximum Relative Light Unit (RLU)) in the *rgs1-1* and *rgs1-2* mutants at 1μM flg22 induction (Fig 1A and S1A Fig). *agb1*-2 and all other mutants including *agb1*-2 showed ~ 80% reduced ROS peak (*P* value < 0.0001; Student's t-test is used to compare to wild type). On the other hand, the *rgs-1*-2 / *gpa1*-4 double mutant had similar ROS levels with *rgs1-2* in line with the previously reported *gpa1*-4 wild type ROS responses to flg22 treatment [7,41] (Fig 1A and S1 Fig).

We extended a role for AtRGS1 in the flg22 response using the intensity-based Ca^2+^ sensor R-GECO1 [42]. We generated stable transgenic Arabidopsis lines in wild type, *rgs1-2* and *bak1-4* backgrounds expressing cytosolic and nuclear localized R-GECO1, which is a red-shifted intensity-based Ca^2+^ reporter. flg22-induced Ca^2+^ signals in wild type, *rgs1-2* and *bak1-4* plants were measured and normalized against the untreated samples (Fig 1B). Ca^2+^ levels represented by fractional fluorescence changes (ΔF/F; the difference between the fluorescence intensity before and after flg22 application/ initial fluorescence intensity) increased from 0 to ~0.7 in wild type (*P* value < 0.001; Student's t-test is used to compare to initial intensity) while ΔF/F was greatly diminished (~0.05) in the *rgs1-2* mutant in response to a 1 min, 1 μM flg22 exposure. *bak1-4* mutants showed an attenuated ΔF/F (~0.3) (*P* value < 0.05; Student's t-test was used to compare to initial intensity) increase consistent with previous findings [43].

### AtRGS1 interacts with BAK1 and BIR1 *in vivo*

**Fig 2:**
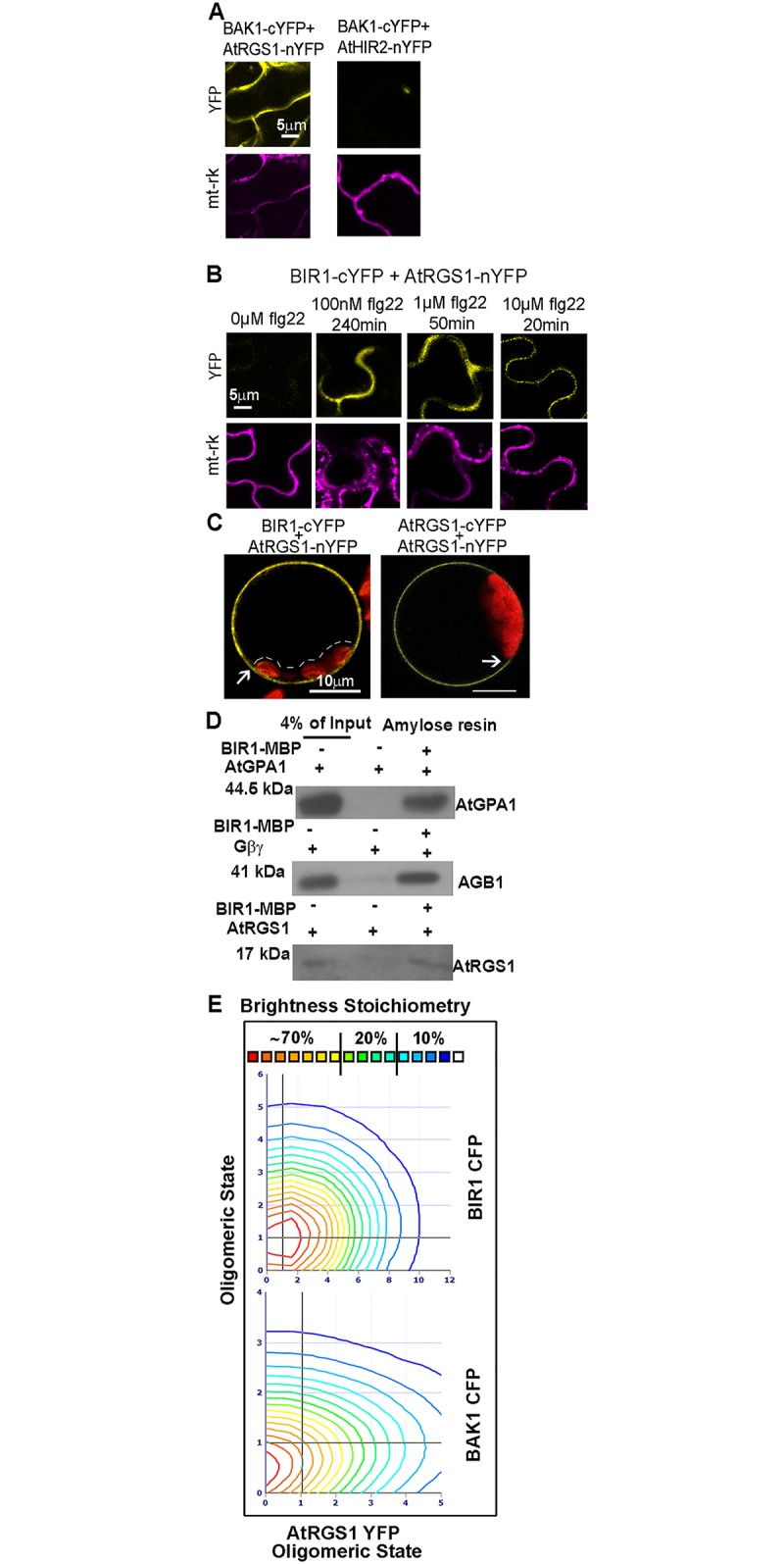
BIR1 interacts with AtRGS1 *in vivo*. **(A)** BAK1 complements AtRGS1 in BiFC. Confocal images of *N*. *benthamiana* cells expressing *BAK1-cYFP* and *AtRGS1-nYFP* showing the putative complementation whereas *BAK1-cYFP* and the negative control *AtHIR2-nYFP* do not complement fluorescence. Yellow signal indicates the YFP complementation results and the purple signal is a transformation reporter, mitochondria protein tagged with mCherry (mt-rk). **(B)** BIR1 complements AtRGS1 in BiFC in a ligand dependent way. Confocal images show constitutive fluorescence complementation of *N*. *benthamiana* cells expressing *BIR1-cYFP* and *AtRGS1-nYFP* at different time points with various flg22 treatments. **(C)** The BIR1-AtRGS1 interaction occurs on the plasma membrane. Confocal images of mesophyll protoplasts expressing *BIR1-cYFP* and *AtRGS1-nYFP or AtRGS1-cYFP* and *AtRGS1-nYFP*. The dashed line shows that no YFP signal is apparent at the chloroplast distal to the plasma membrane. The arrow points to clear demarcation of plasma membrane signal from cytoplasm. **(D)** The interaction between BIR1 and AtRGS1 and the heterotrimeric subunits is through direct contact. Immunoblots of protein that were retained on a BIR1 kinase domain-maltose-binding protein (BIR1-MBP)-amylose resin are detected by probing with antibodies directed against AtGPA1, AGB1, and AtRGS1 as indicated. Details are described in Materials and Methods **(E)** Stoichiometry histogram from cross N&B analysis between AtRGS1 and RLKs. The x -axis shows AtRGS1-YFP oligomeric state whereas the y -axis represents the RLKs’ oligomeric state (top: BIR1-CFP; bottom BAK1 CFP); 1 is monomer and 2 is dimer. Relative proportions of the oligomeric states are demonstrated with red to blue colored lines. Red and yellow lines form about ~70% of the pixels; green ones are ~20% of the pixels and blue lines are ~10% of pixels in the interactions indicated on the axes. Black line across the graph is placed where the monomer to monomer interaction is read.

The LRR-RLK FLS2 interacts with XLG2 in flg22-based signaling [13]. BAK1 is the co-receptor of FLS2 and also interacts with BAK1-interacting receptor-like kinase (BIR1) which coordinates with AGB1 in the cell death response associated with innate immunity [41]. G protein signaling is directly activated by BAK1 in response to flg22 [16]. We determined if AtRGS1 physically interacts *in vivo* with two receptors partnering with BAK1, FLS2 and BIR1, using bimolecular fluorescence complementation (BiFC) over time. BAK1 and AtRGS1 interacted *in vivo* (Fig 2A); however, fluorescence complementation of AtRGS1 with FLS2 was not observed in the presence or absence of flg22 indicating that either FLS2 does not interact directly with AtRGS1 or that the interaction is not detectable using this technique at this concentration and time course range (S2A Fig). Fluorescence complementation between BIR1 and AtRGS1 was flg22 dependent in *N*. *benthamiana* cells (Fig 2B). Several flg22 exposure times and concentrations demonstrated this strict flg22 dependence. A final control for specificity was performed by placing the n-YFP domain at the N-terminus of BIR1 (extracellular) and paired with AtRGS1 tagged with c-YFP at the C terminus (cytoplasmic). In this conformation, fluorescence complementation did not occur as expected (S2B Fig).

The limitations of BiFC are that fluorescence complementation is irreversible and is dependent on the starting orientation of the YFP split halves. Therefore, a positive BiFC result is unable to provide information about the dynamics of the interaction, the number and kinds of intermediate states, or the direction of movement of any of these intermediate states. Nonetheless, the flg22 dependency of this fluorescence complementation (Fig 2B) informs us that flg22 sets in motion one or more reactions that include an interaction state between BIR1 and AtRGS1.

The presence of a large vacuole in many plant cells confines the cytosol to a thin layer at the cell periphery making it difficult to distinguish fluorescence signal between cell membrane and cytosolic subcellular location [44]. However, sufficient resolution is obtained using mesophyll protoplasts. The technical distinction of subcellular location is elaborated by Wolfenstetter and co-workers [45] with examples of signals in the cytoplasm, on the tonoplast, and on the plasma membrane based on the position of the signal relative to the chloroplast. As shown in Fig 2C, the AtRGS1-BIR1 is clearly located on the plasma membrane similar to the AtRGS1 homodimer (arrows, Fig 2C). Fluorescent signal was never observed in the cytoplasm as indicated by the lack of signal at the tonoplast edge of the chloroplasts (brackets, Fig 2C).

To determine if AtRGS1 and BIR1 proteins directly share a protein interface, we tested interaction of the pure recombinant proteins *in vitro* using co-immunoprecipitation. The BIR1 kinase domain alone (the extracellular and TM domains deleted) was tagged with maltose-binding protein (MBP) at the N terminus and expressed in *E*. *coli*. The MBP-tagged BIR1 kinase domain was immobilized with amylose beads. Recombinant AGB1, AtGPA1 or the cytoplasmic RGS domain of AtRGS1 was loaded on the column and after extensive washing, complexes were eluted with maltose. Immunoblot analysis confirmed that AGB1, GPA1 and AtRGS1 directly interacted with BIR1, which is previously reported to interact with GPA1 and AGG1 and 2 in yeast two hybrid and BiFC assays [15] (Fig 2D) suggesting the existence of a large RLK(s)/AtRGS1/G protein signaling complex *in vivo*.

Next, we performed cross number and molecular brightness (N&B) analysis [36,37] to determine the stoichiometry of the AtRGS1-RLK complexes. Each protein was tagged with a different fluorophore and transiently expressed in pairs, AtRGS1-YFP and BIR1-CFP; AtRGS1-YFP and BAK1-CFP, in *N*. *benthamiana*. A time stack of raster-scanned images was obtained using a Zeiss 880 (http://www.zeiss.com/) microscope with 458-nm and 514-nm argon lasers that were tuned to excite CFP and YFP, respectively (see Material and Method). In addition, cells expressing only AtRGS1-YFP or RLK-CFP and lines expressing only YFP were used to set the background fluorescence and to measure monomer brightness. The mean and the variance of the intensity distribution at each pixel were collected in order to determine the number (N) and brightness (B) of the particles and the cross-correlation between the two channels (YFP and CFP). At each pixel the SimFCS Software Analysis determined the average number of proteins in in a complex. The stoichiometry contour map generated from cross N&B analysis (Fig 2E) shows the proportion of the different complexes. Red and yellow lines representing AtRGS1 in the complex represented about ~70% of the pixels; the red line at (1, 1 = monomer to monomer) shows that a high proportion of monomeric AtRGS1-YFP is bound to monomeric BIR1- CFP (n = 6) or to BAK1-CFP and (n = 3). SimFCS Software Analysis showing color-coding of the cross brightness of the RLK-CFP/AtRGS1-YFP in representative samples is depicted in S3 Fig. Blue represents RLK monomer binding AtRGS1 monomer, while green represents AtRGS1 homodimer binding RLK monomer. Cross N&B results revealed that AtRGS1 is able to bind the monomers of RLKs *in vivo*.

### AtRGS1/G protein LRR RLK complexes are dynamic

**Fig 3:**
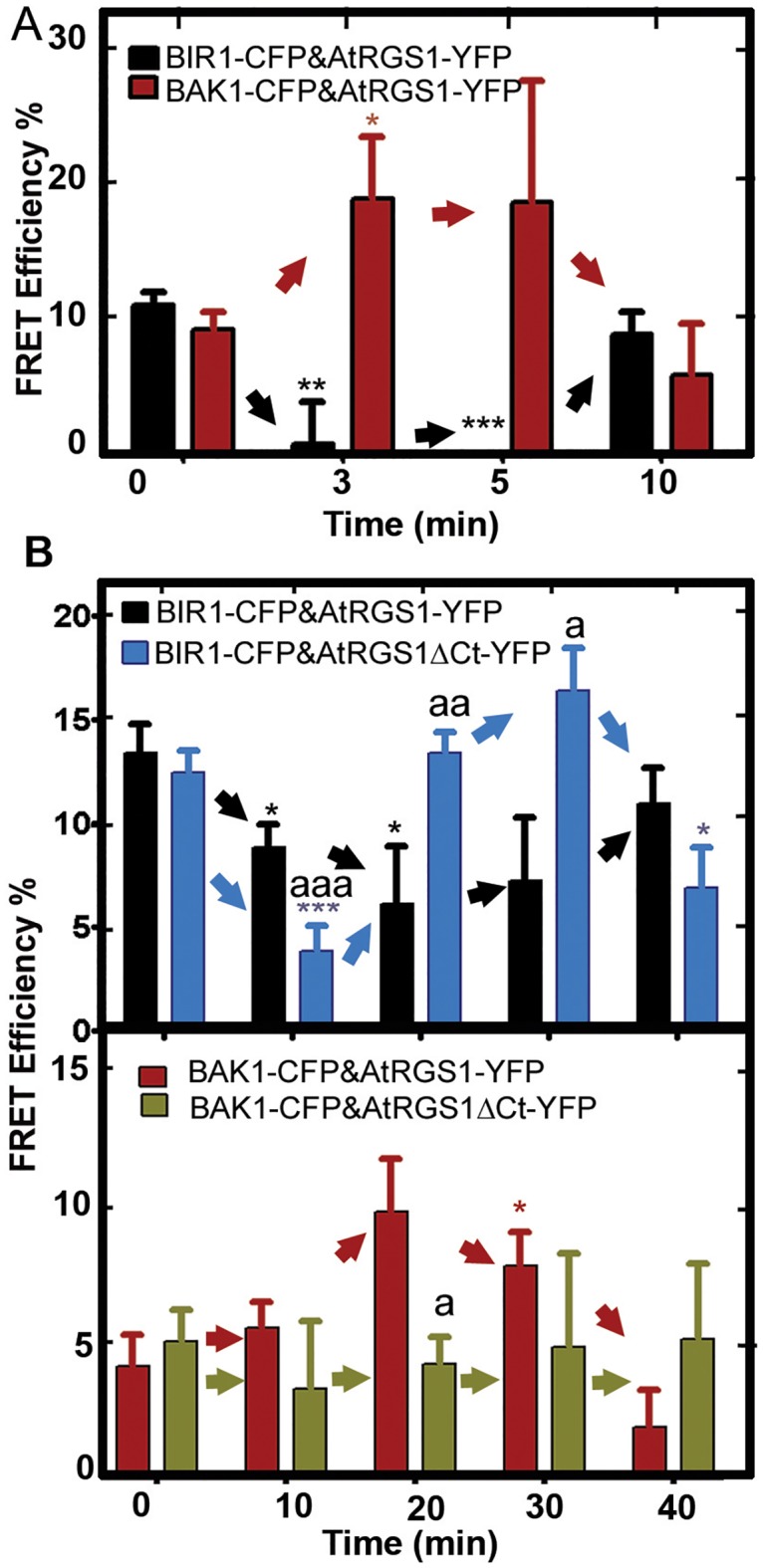
flg22-induces changes in AtRGS1/ RLKs complex dynamics. **(A)** flg22 rapidly causes AtRGS1 to move away from BIR1 and toward BAK1. FRET analysis (acceptor photobleaching) of *N*. *benthamiana* cells expressing BAK1-CFP, BIR1-CFP and AtRGS1-YFP in the presence of flg22 (1μM) at the indicated time points is shown. Error bars represent standard error of the mean (SEM) of regions of interests (ROIs) n = 3 to 16. **(B)** Top: The dynamics of the BIR1-AtRGS1 is modulated by the phosphorylated C-terminal domain. FRET efficiency in *N*. *benthamiana* cells expressing BIR1-CFP AtRGS1-YFP or AtRGS1ΔCt-YFP in the presence of a low concentration of flg22 (100 nM) is shown over time. Error bars represent SEM of ROIs (n = 4 to 23). Bottom: BAK1 interaction requires the carboxy-terminal domain of AtRGS1. FRET efficiency in *N*. *benthamiana* cells expressing BAK1-CFP and AtRGS1-YFP or AtRGS1ΔCt-YFP in the presence of a low concentration of flg22 (100 nM) is shown over time. Error bars represent SEM of ROIs (n = 3 to 17). All experiments were repeated at least two times. It is important to note that panel A shows results of flg22 at a moderate concentration (1 μM) and panel B show the results of flg22 at a low concentration (100 nM), hence the different time courses. Student's t tests were conducted to compare the FRET Efficiency % of flg22 treated leaves at the indicated time points to 0 min. ***, Student's t test significant at *P* value < 0.0001; **, Student's t test significant at *P* value < 0.01; *, Student's t test significant at p < 0.05. AtRGS1-YFP vs AtRGS1ΔCt-YFP interactions in B are shown by a letter: aaa, aa or a.

We showed that AtRGS1 is involved in the ROS burst (Fig 1A). Therefore, we tested the change in physical distances between AtRGS1, BIR1, and BAK1 (Fig 3) within the time frame of ROS production (S4 Fig). After 3 min of flg22 (1 μM) addition, the FRET efficiency between AtRGS1 and BIR1 dropped to zero then returned to baseline over 10 minutes (Fig 3A) indicating a major but transient conformational change and suggesting that AtRGS1 and BIR1 separated from each other. Mirroring the AtRGS1-BIR1 proximity kinetics, AtRGS1 and BAK1 appeared to approach each other as indicated by the increased FRET efficiency.

A longer time course and a lower flg22 demonstrated that the change in physical distances between AtRGS1, BIR1, and BAK1 are concentration and time dependent. At a low concentration of flg22 (100 nM, Fig 3B), the same dynamics occurred as at 1 μM (cf. Fig 3A with 3B) although the maximum changes occurred later (10 min vs. 3 min for BIR1 and 20 min vs. 3 min for BAK1) (Fig 3B). BAK1 phosphorylates AtGRS1 at its C-terminal tail [16]. To determine if the C-terminal tail of AtRGS1 is needed for flg22-induced changes in the dynamics of the RLK and AtRGS1 complex, an AtRGS1 protein lacking this region (AtRGS1-ΔCT, Fig 3B) [16,20] was tested. flg22 failed to induce a conformational change between AtRGS1-ΔCT and BAK1 (Fig 3B Bottom) while AtRGS1-ΔCT still interacted with BIR1 albeit returning to baseline more rapidly (Fig 3B Top), positing the following order of conformational changes within this complex of AtRGS1 and RLKs: **1)** flg22 binding to the FLS2/BAK1 heterodimer promotes the loss of BAK1 consequently leaving FLS2 to be degraded [46]. **2)** BAK1 then displaces BIR1 from the BIR1/AtRGS1 complex components (Fig 3A) and, **3)** based on the *in vitro* data [16] and the ΔCT data of Fig 3, may phosphorylate AtRGS1.

## Discussion

Our results suggest that LRR RLKs operate in the canonical G protein complex via dynamic interactions with AtRGS1 that overlap the time span that the ROS burst and calcium release responses occur. The AtRGS1/BAK1/FLS2/BIR1 receptor kinase complex mechanism described here encompasses ligand (flg22) to cellular output (i.e. ROS burst) over a time scale resolved from seconds to minutes. The spatial and temporal resolution enabled assembly of an orchestrated order of conformational relationships between the key signaling molecules.

**Fig 4:**
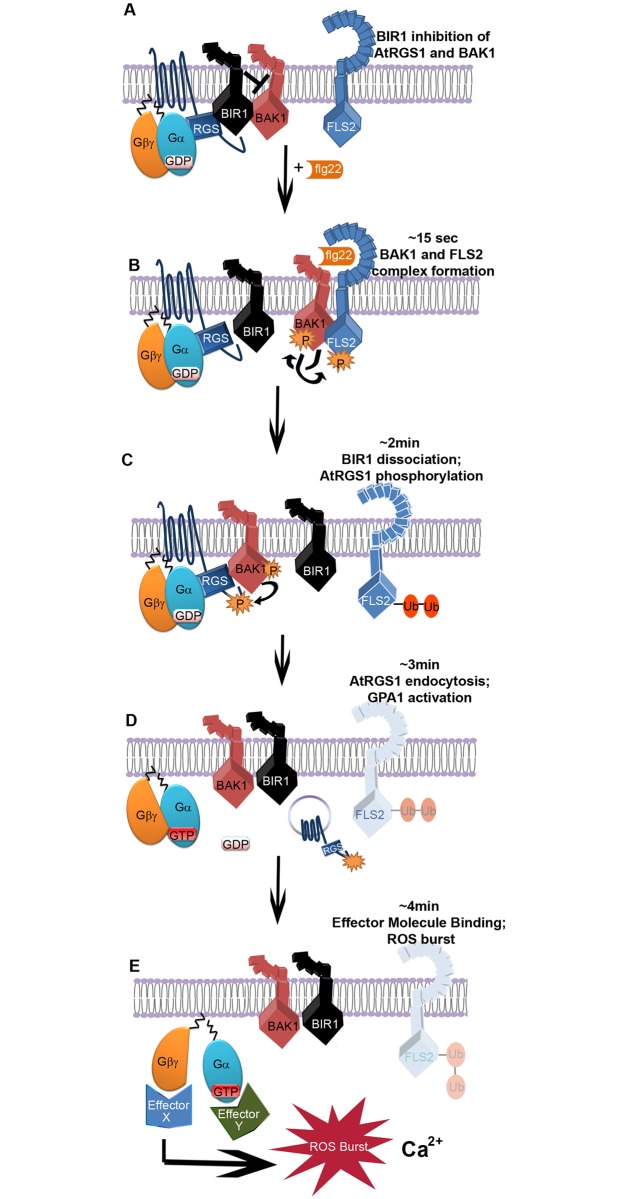
Simplified scheme of direct activation of G signaling by an RLK. **(A)** At resting state, BIR1, BAK1, and AtRGS1 (RGS1) are in complex with the GαGβγ. **(B)** At ~15 sec, flg22 binds FLS2 leading to BAK1-FLS2 dimerization with trans- and auto- phosphorylation. In the meantime BIR1 is triggered to move away by an unknown component. **(C)** At ~2 min, BAK1 phosphorylates AtRGS1 and thus prevents the RGS1-BIR1 coming in close proximity before everything returns to its base line. FLS2 becomes poly-ubiquitinated and destined for degradation as shown in faded form in D and E. **(D)** At ~3 min, phosphorylated RGS1 is endocytosed, physically uncoupling from GαGβγ. Gα is now able to spontaneously exchange nucleotides leading to activation of Gα^GTP^ and Gβγ*. Endocytic RGS1 is shown in the vesicle. **(E)** t ~4 min, Gβγ initiates the ROS production. Most of the ROS production is through activated Gβγ. The nature of effector X and Y are not known. The scheme does not depict all the transient states, but highlights rate-limiting components and conveys the control points along the time course of ligand activation.

Based on the dynamic data here in addition to the published data, we propose that the order of partner selection over time after flg22 induction follows the scheme summarized in Fig 4: **1)** flg22 binding induces nearly instantaneous dimerization between FLS2 and BAK1 [47,48] with both co-receptors participating in flg22 recognition [49]. **2)** Phosphorylated BAK1 interacts with and enables AtRGS1 to move away from BIR1 (Fig 3). **3)** AtRGS1 becomes phosphorylated leading to its endocytosis thus leading to de-repression by permitting AtGPA1 to exchange GDP for GTP **4)** The G protein complex becomes dissociated thus AGB1 interacts with its effector proteins, some leading to changes in ROS and calcium.

There are three possible mechanisms how the interaction of the AtRGS1 with BAK1 and BIR1 alters function of the system: **1)** Interaction of AtRGS1 alters the conformation to its Gα substrate and by doing so is no longer able to GAP it, thus Gα self activates [16]. **2)** flg22 directly inhibits AtRGS1 catalytic GAP activity, although this mechanism is unlikely since flg22 acts extracellularly while the RGS catalytic domain is cytoplasmic. Nonetheless, this mechanism remains a possibility since we have not yet tested the GAP activity of AtRGS1 in the presence of RLKs. **3)** The adaptor mediating AtRGS1 endocytosis requires AtRGS1 to interact with BAK1 before it recruits clathrin. To test this hypothesis, we need to know the adaptor. There are no β-arrestins homologs in plant cells but there are candidate arrestin-fold proteins [50] to be tested in follow-on studies. We do not exclude the possibility that the role of AtRGS1 phosphorylation in the G protein activation mechanism is also required for adaptor recognition.

FLS2 interacts with XLG2, and in the presence of flg22, the canonical G signaling components that were originally held inactive by AtRGS1 become activated by BAK1. We speculate that release of AtGPA1 from the canonical heterotrimeric complex upon removal of AtRGS1 allows association of Gβγ with XLG2. This newly-formed XLG- Gβγ becomes the substrate for BIK1 [51,52], leading to XLG2 phosphorylation and ultimately degradation of BIK1 [13]. Because BIK1 phosphorylation of the AtRboh complex is important for the flg22-induced ROS burst, the atypical G protein pathway provides an additional negative feedback mechanism on ROS generation. Therefore, both the canonical G protein and the atypical G protein pathways act as governors of ROS production.

## Supporting information

S1 Fig *rgs1-2* mutants have partial impairment in ROS burst. flg22-induced, FLS2-dependent ROS production is greatly attenuated in the *rgs1* and *agb1* null mutants. ROS, reported as Relative Luminescence Units (RLU) in leaf disks treated with flg22 (1 μM) from 5-week-old seedlings was measured over time. Error bars are SEM and sample size *n* = 6–15.
(TIF)

S2 Fig BiFC analysis between FLS2-cYFP and AtRGS1-nYFP *in N*. *benthamiana*. **(A)** Confocal images of *N*. *benthamiana* cells expressing *FLS2-cYFP* and *AtRGS1-nYFP* to analyze the putative complementation in the presence of the indicated concentrations of flg22 and captured at the indicated times. **(B)** Three additional negative controls of BiFC assays for BIR1 indicate that the positive flg22-induced BIR1-RGS1 interaction (Fig 2) is specific and dependent on the BiFC-tagged conformation. Top rows in (A) and (B) show no fluorescence complementation between these test pairs. Bottom row. Transformation control is shown. The presence of mCherry indicates that these cells are expressing the test constructs.
(TIF)

S3 Fig SimFCS software analysis shows color-coding of the cross brightness of the RLK-CFP/AtRGS1-YFP expressing *N*. *benthamina* cells. Blue represents RLK monomer binding AtRGS1 monomer, while green represents AtRGS1 homodimer binding RLK monomer.
(TIF)

S4 Fig *N*.*benthamiana* ROS burst starts within 6 minutes of 100 nM flg22 application. flg22-induced ROS production in *A*. *thaliana* and *N*. *benthamiana* plants over 50 min. Error bars are SEM and sample size n = 13–20.
(TIF)

## Abbreviations

AGB1: Arabidopsis Gβ subunit
AtGPA1: *Arabidopsis thaliana* Gα subunit
AtRboh: *Arabidopsis thaliana* respiratory burst oxidase homologs
AtRGS1: *Arabidopsis thaliana* Regulator of G Signaling 1
BAK1: BRI1-associated Kinase 1
BIR1: BAK1-interacting receptor-like kinase1
flg22: flagellin 22-amino acid peptide
FLS2: flg22 receptor
FRET: Förster Resonance Energy Transfer
LRR RLK: leucine-rich repeat receptor–like kinases
PAMP: pathogen-associated molecular patterns
ROS: Reactive Oxygen Species
XLG: extra-large G protein Gα subunit
