# Supplementary figures and images for: Ligand-induced dynamics of heterotrimeric G protein-coupled receptor-like kinase complexes
Source: PLoS One. 2017 Feb 10;12(2):e0171854. doi: 10.1371/journal.pone.0171854 (PMC5302818; doi:10.1371/journal.pone.0171854)

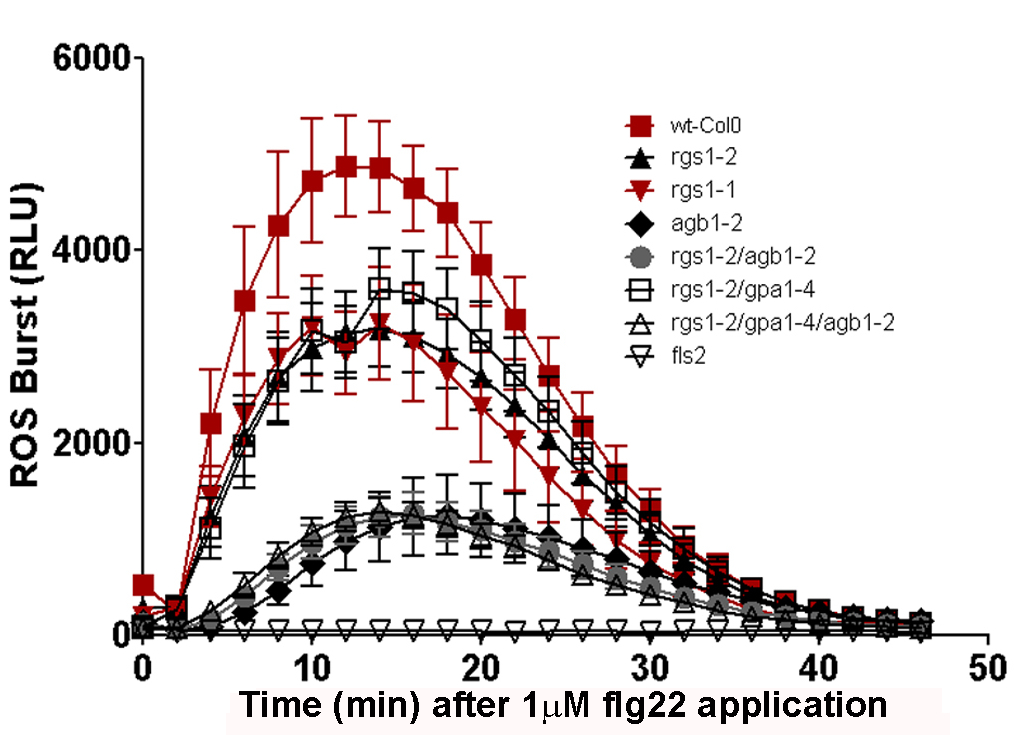

Supplement: S1 Fig — flg22-induced, FLS2-dependent ROS production is greatly attenuated in the rgs1 and agb1 null mutants. ROS, reported as Relative Luminescence Units (RLU) in leaf disks treated with flg22 (1 μM) from 5-week-old seedlings was measured over time. Error bars are SEM and sample size n = 6–15. (TIF) [file pone.0171854.s001.tif]

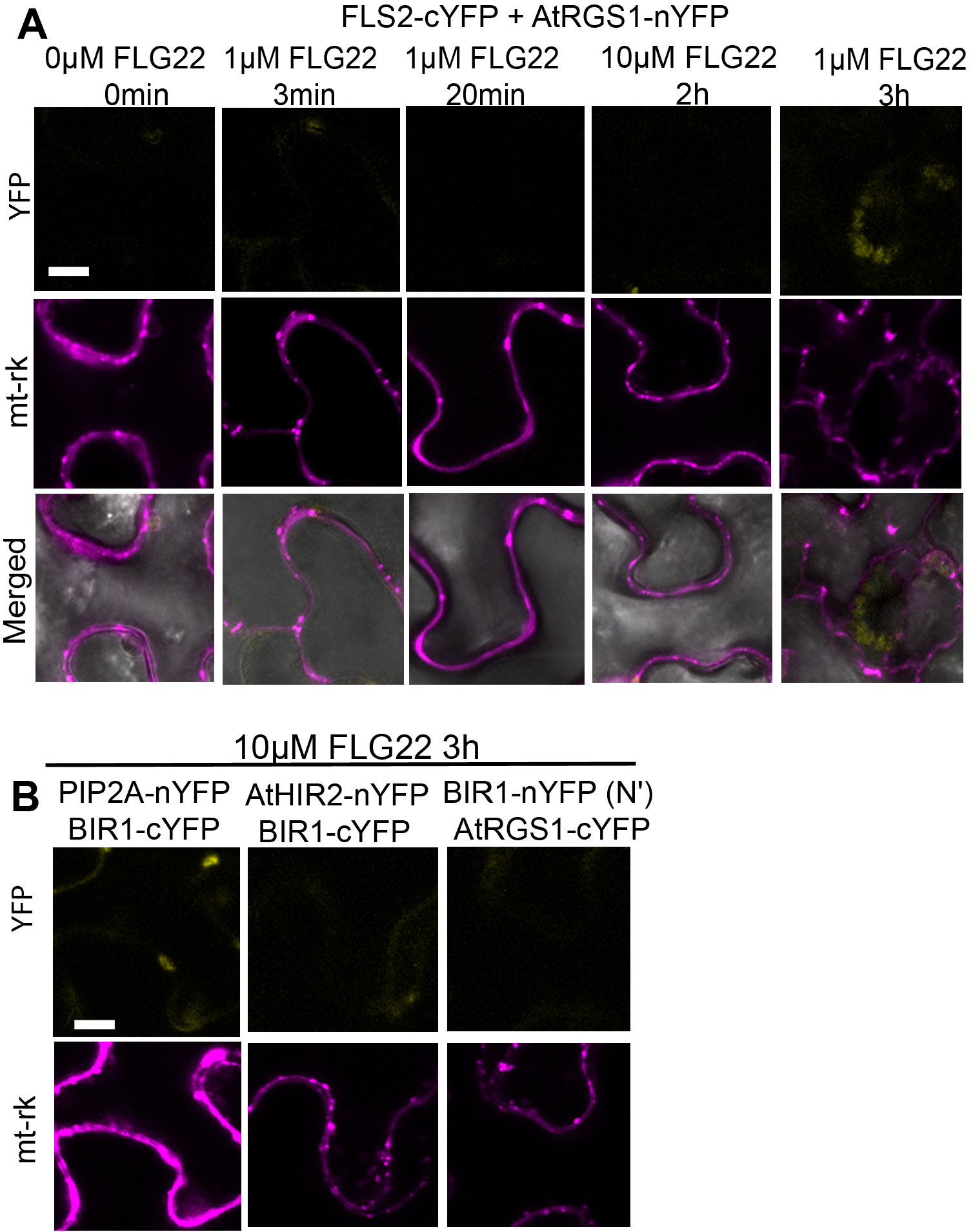

Supplement: S2 Fig — (A) Confocal images of N. benthamiana cells expressing FLS2-cYFP and AtRGS1-nYFP to analyze the putative complementation in the presence of the indicated concentrations of flg22 and captured at the indicated times. (B) Three additional negative controls of BiFC assays for BIR1 indicate that the positive flg22-induced BIR1-RGS1 interaction (Fig 2) is specific and dependent on the BiFC-tagged conformation. Top rows in (A) and (B) show no fluorescence complementation between these test pairs. Bottom row. Transformation control is shown. The presence of mCherry indicates that these cells are expressing the test constructs. (TIF) [file pone.0171854.s002.tif]

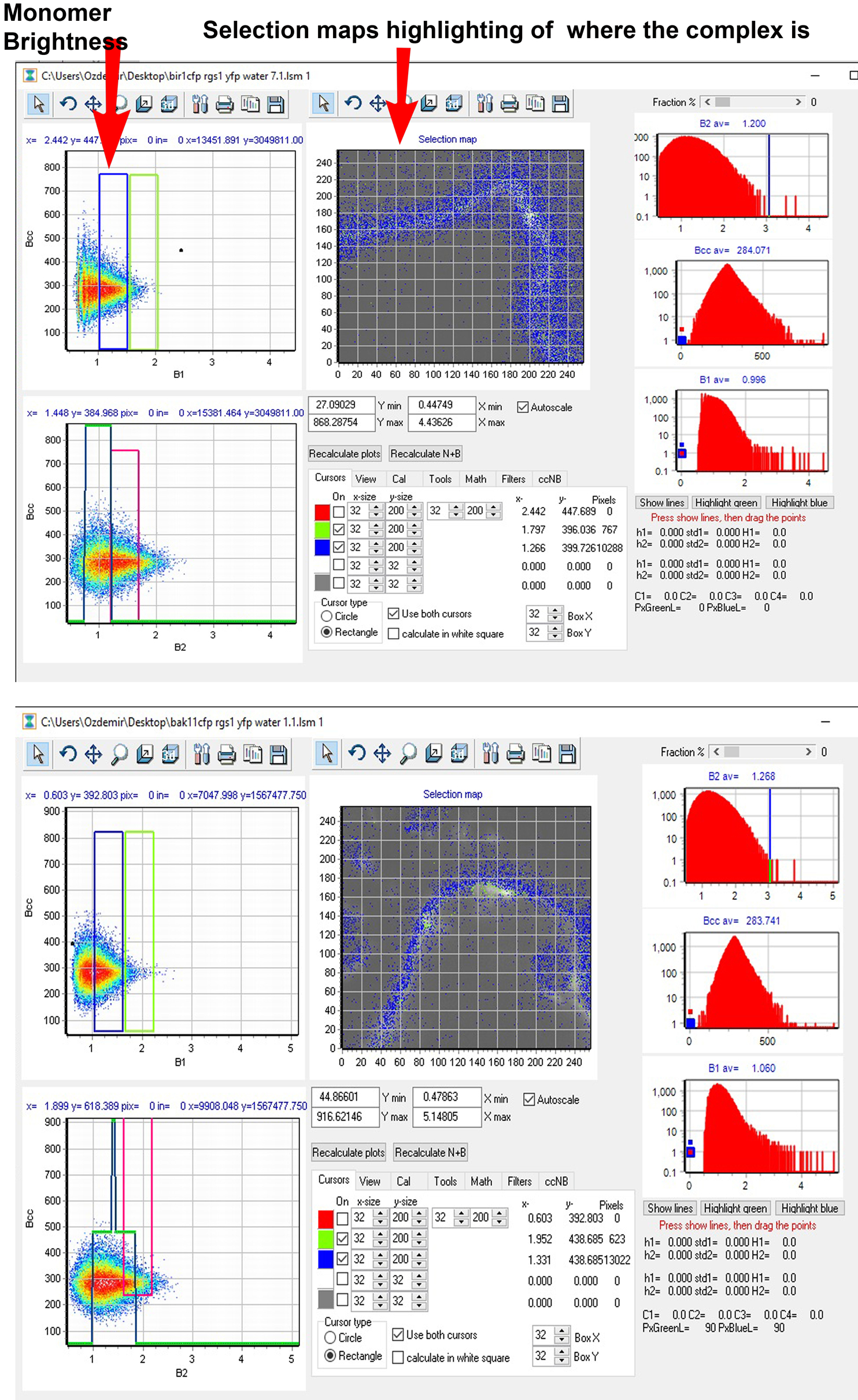

Supplement: S3 Fig — Blue represents RLK monomer binding AtRGS1 monomer, while green represents AtRGS1 homodimer binding RLK monomer. (TIF) [file pone.0171854.s003.tif]

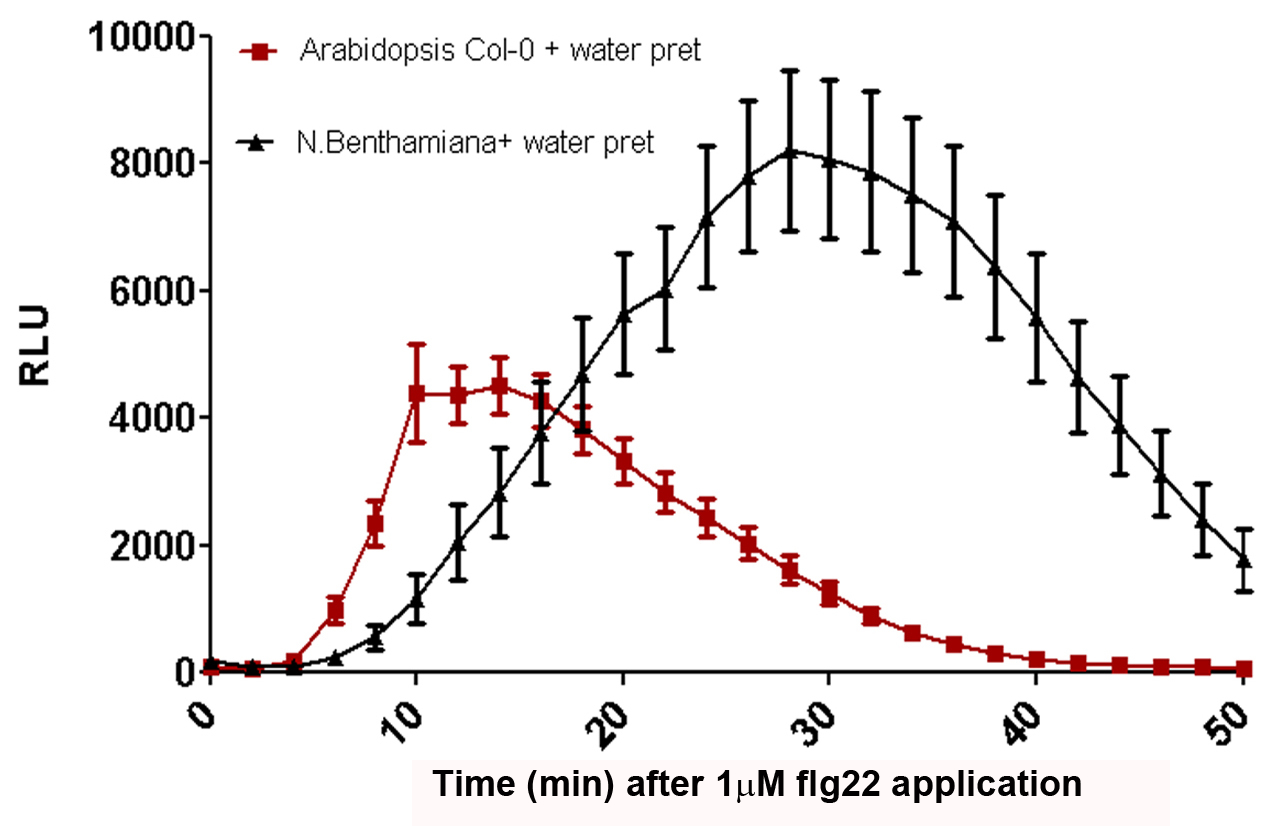

Supplement: S4 Fig — flg22-induced ROS production in A. thaliana and N. benthamiana plants over 50 min. Error bars are SEM and sample size n = 13–20. (TIF) [file pone.0171854.s004.tif]
